# Supplementary material for: Integrated Analysis of the Metabolome and Transcriptome During Apple Ripening to Highlight Aroma Determinants in Ningqiu Apples
Source: Plants (Basel). 2025 Apr 9;14(8):1165. doi: 10.3390/plants14081165 (PMC12030433; doi:10.3390/plants14081165)
Supplement: Supplementary file 1 [file plants-14-01165-s001.zip › plants-3544989-supplementary/Supplementary material/Supplementary material 1.pdf]

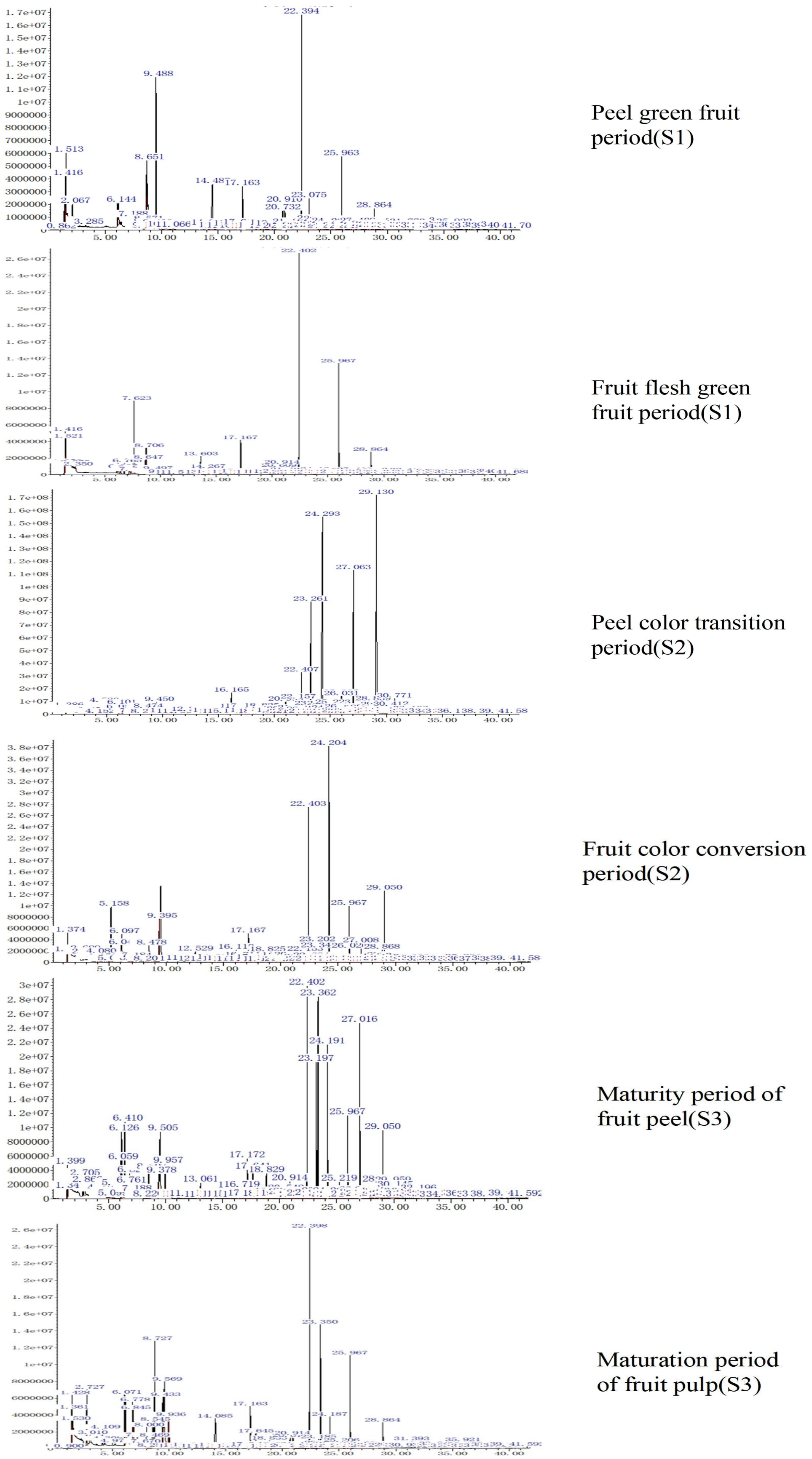


**Figure S1 Aroma ion spectra of fruit peel and flesh during the ripening process, including the green fruit stage, color transition stage, and ripening stage.**

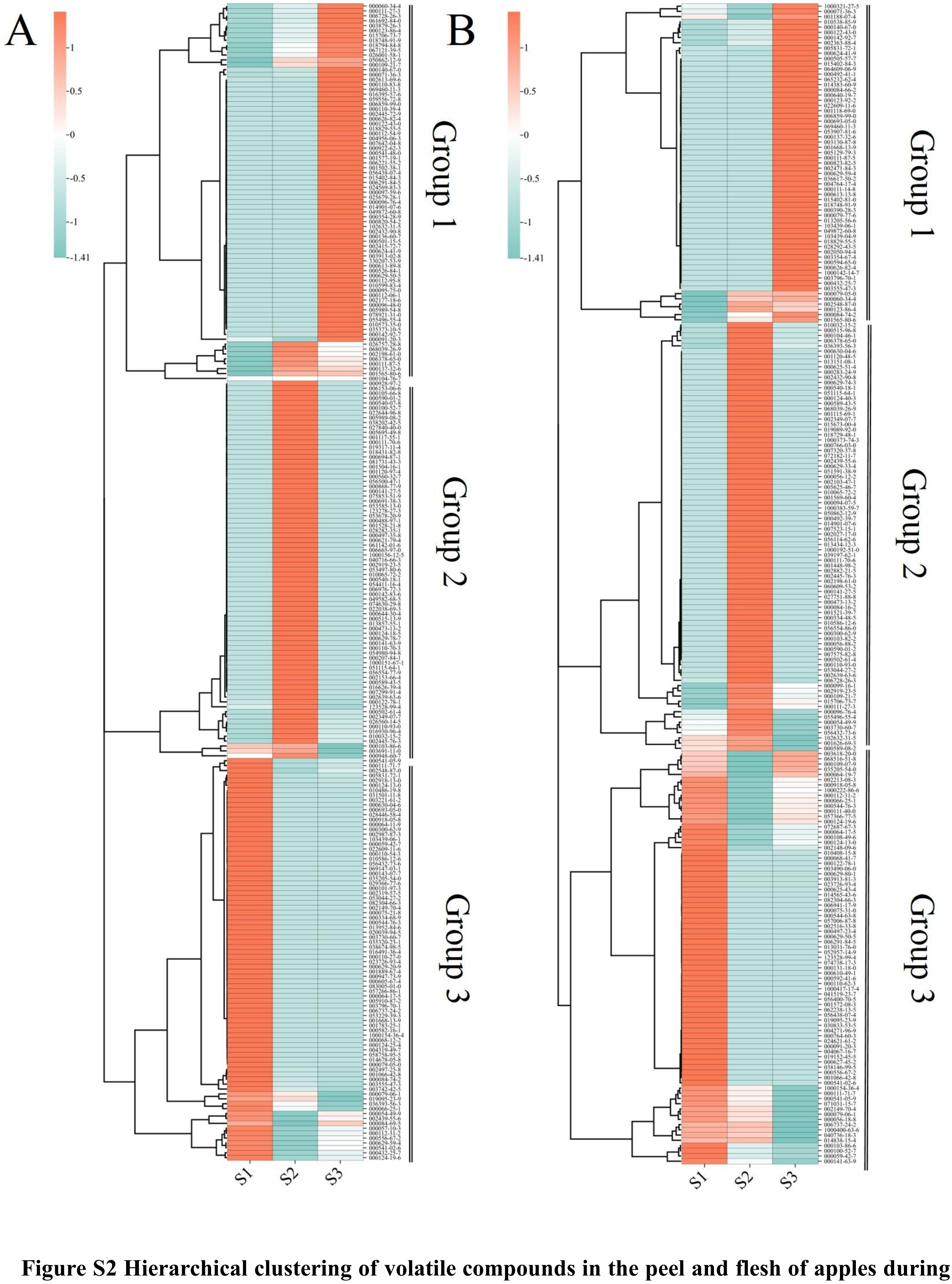


**Figure S2 Hierarchical clustering of volatile compounds in the peel and flesh of apples during ripening. (A) Peel. (B) Fruit pulp.Note: The compound names corresponding to the CAS numbers are provided in Supplementary Materials Table 1 and Table 2. Data are presented as**

**means, with n = 3, and were normalized prior to analysis and plotting.**

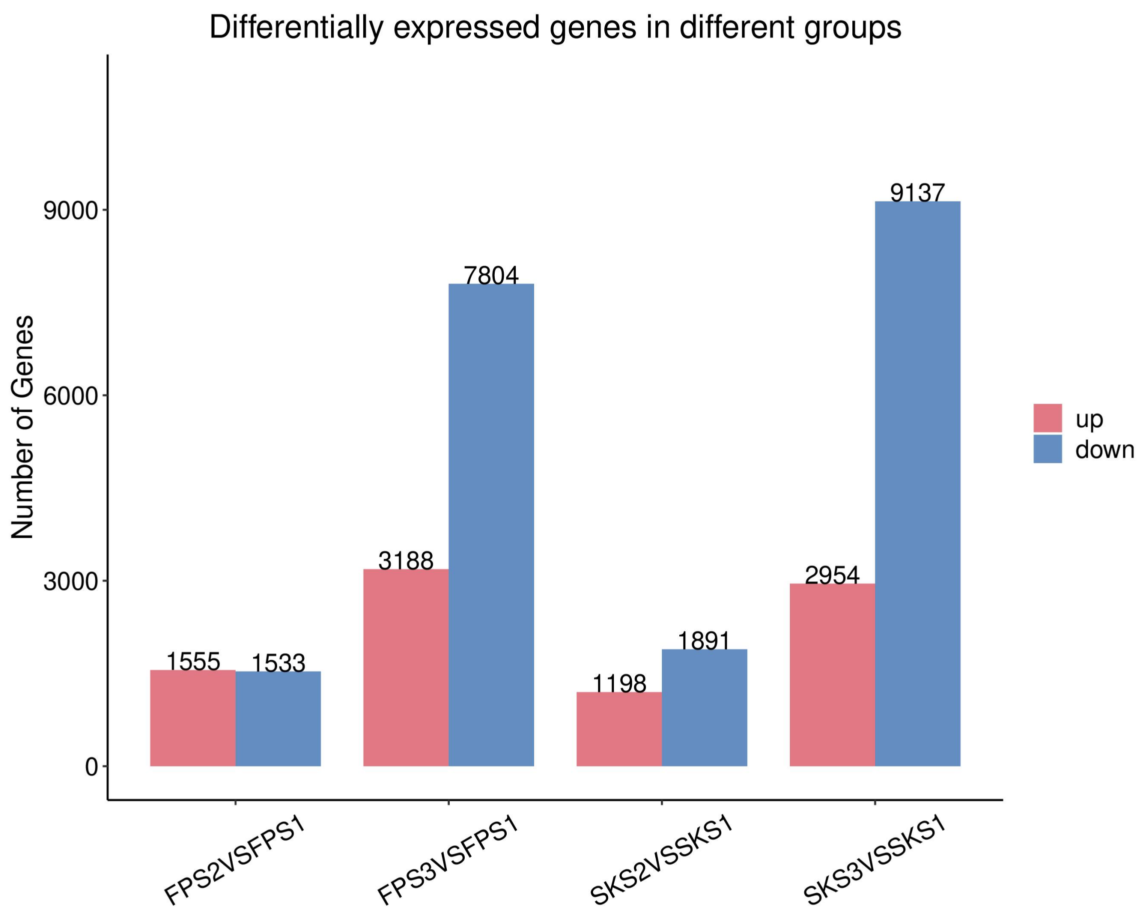


**Figure S3 Differentially expressed genes. FP represents flesh, SK represents skin, S1 represents green fruit stage, S2 represents color transformation stage, and S3 represents maturity stage.**

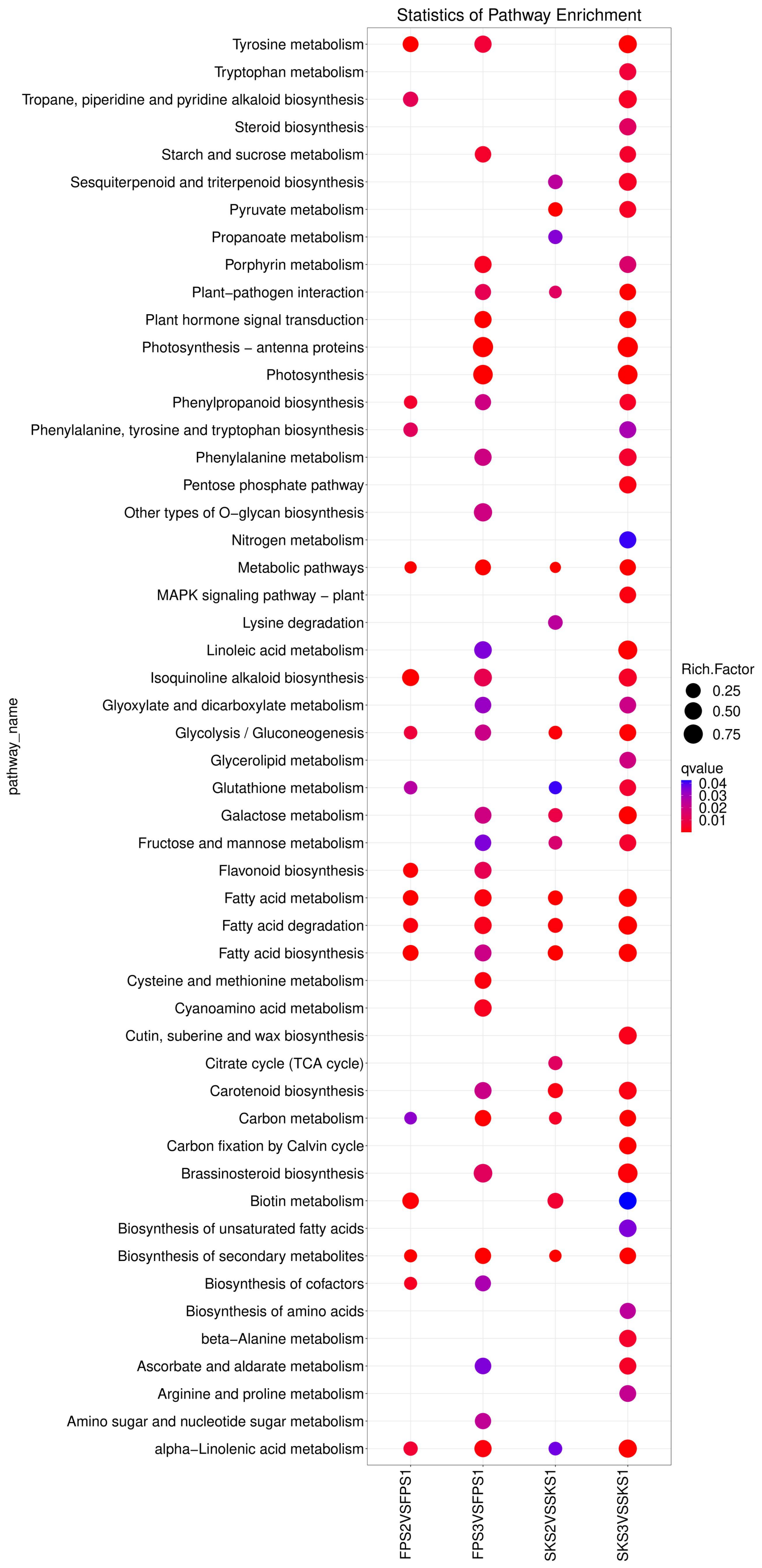


**Figure S4 KEGG enrichment analysis of differentially expressed DEGs in four groups.**

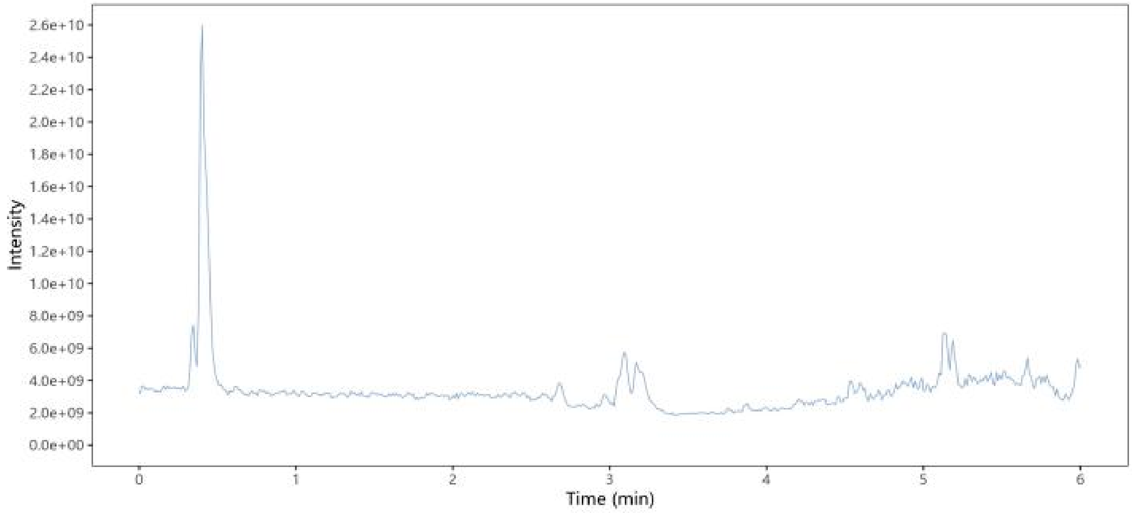


**Figure S5 TIC plot of positive ionization mode for UHPLC-OE-MS detection of QC samples**

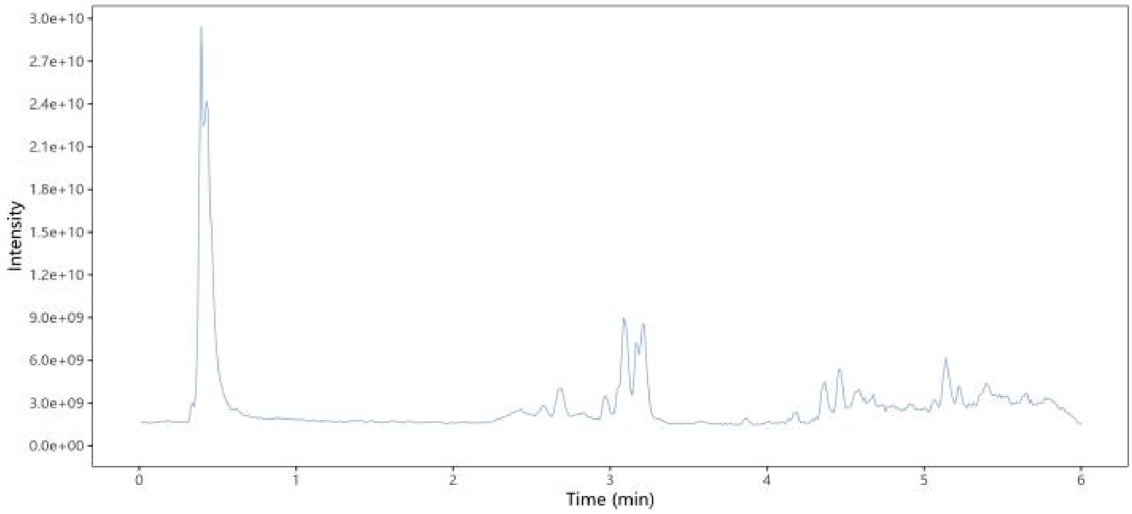


**Figure S6 TIC plot of negative ion mode for QC sample UHPLC-OE-MS detection**

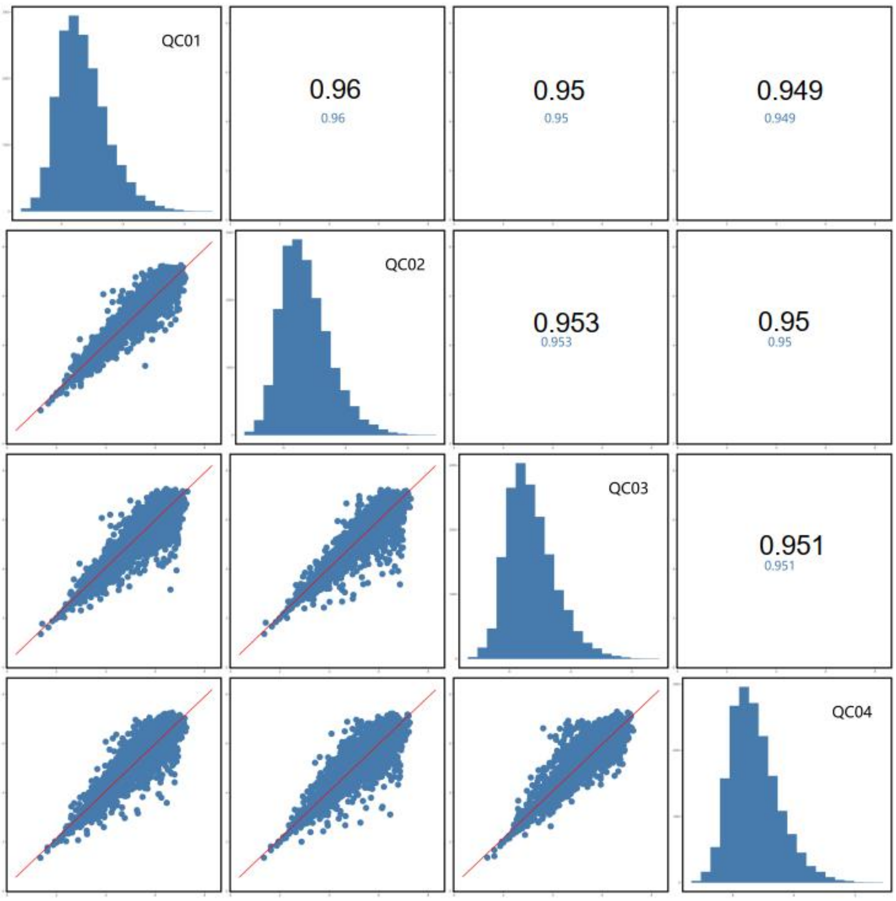


**Figure S7 Correlation between QC samples**

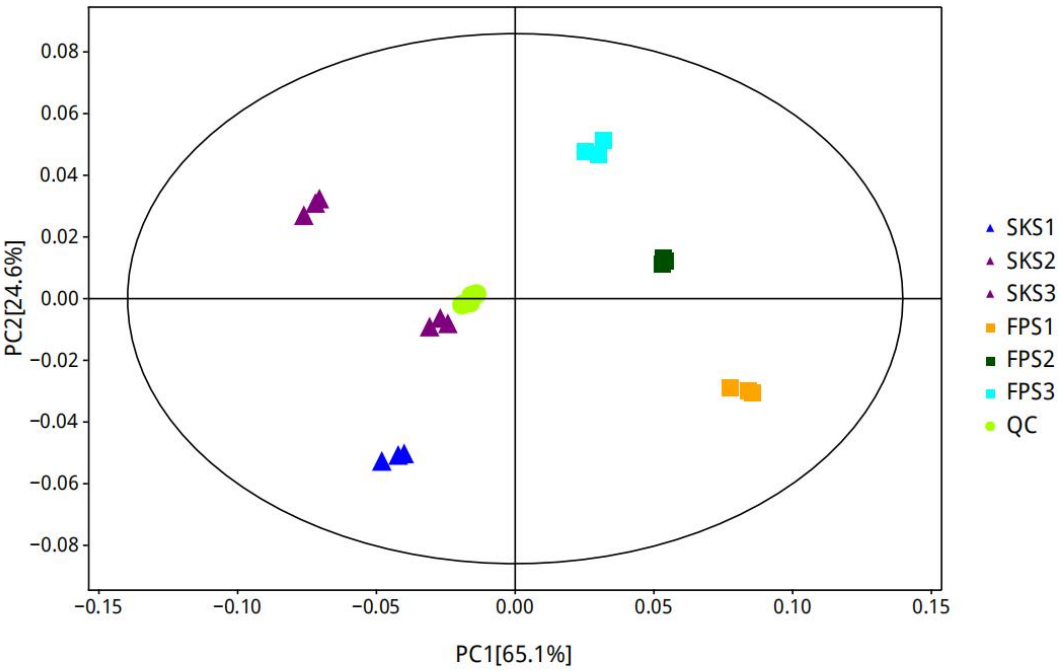


**Fig.S 8 PCA analysis of samples from 3 developmental stages of peel and pulp (QC).**
**FP represents flesh, SK represents skin, S1 represents green fruit stage, S2 represents color transformation stage, and S3 represents maturity stage.**

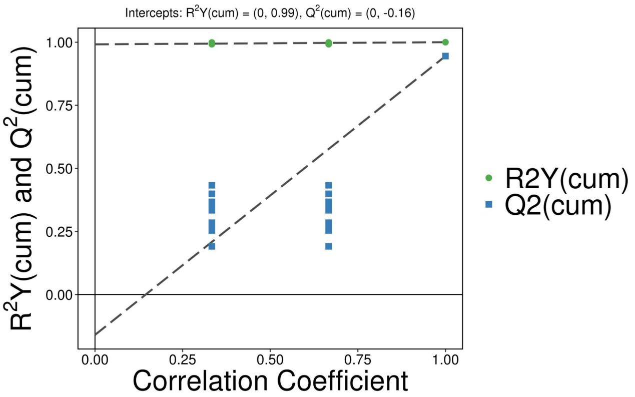


**FPS2vsFPS1**

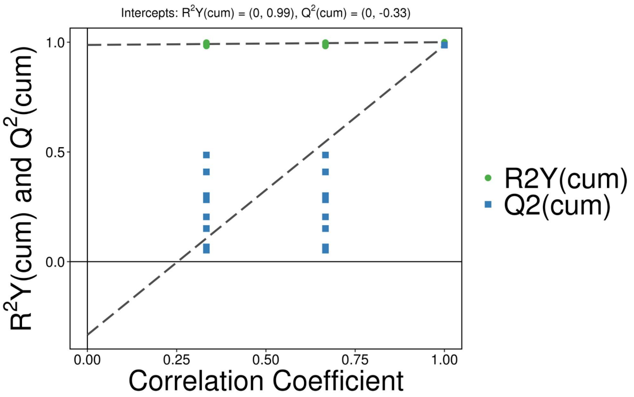


**FPS3vsFPS1**

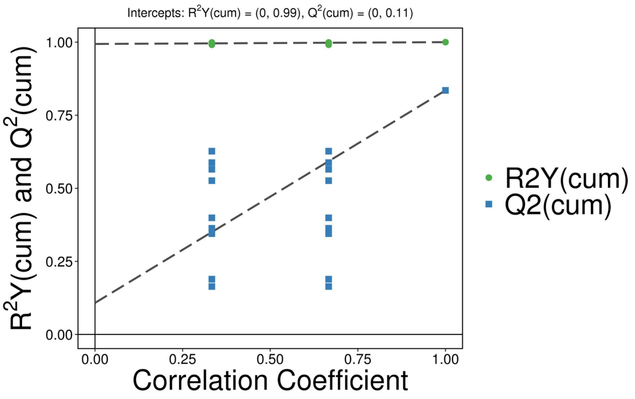


**SKS2vsFPS1**

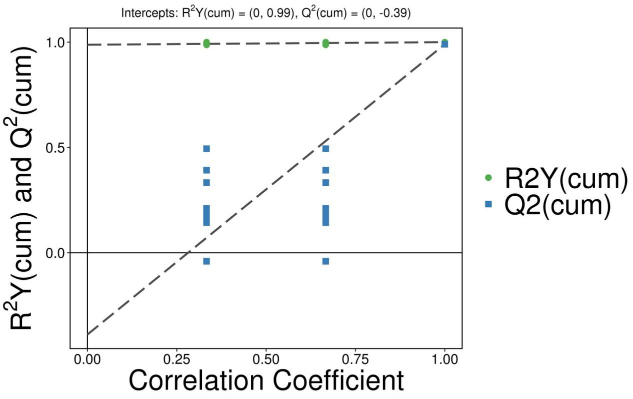


**SKS3vsFPS1**

**Fig. S9 Dot plot of replacement test results for OPLS-DA model (n=200).FP represents flesh, SK represents skin, S1 represents green fruit stage, S2 represents color transformation stage, and S3 represents maturity stage.**

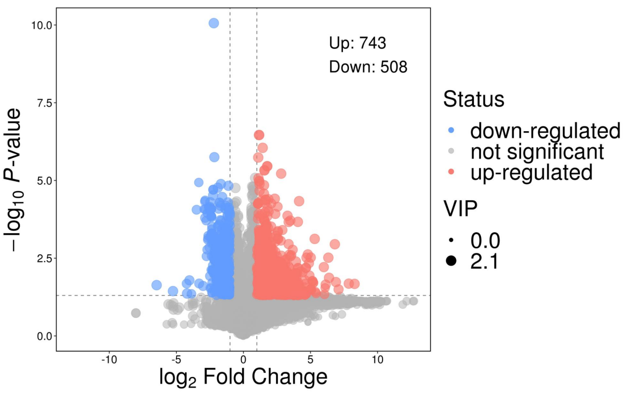


**FPS2vsFPS1**

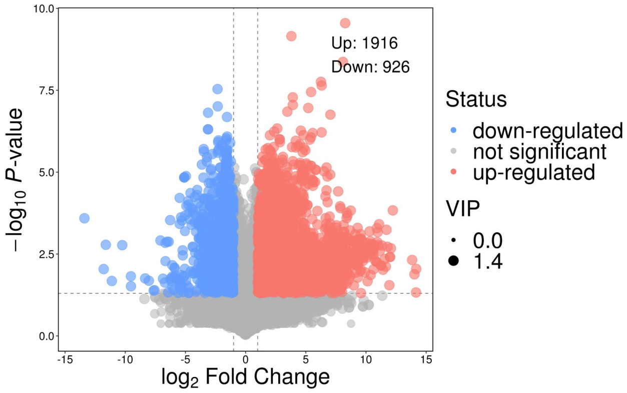


**FPS3vsFPS1**

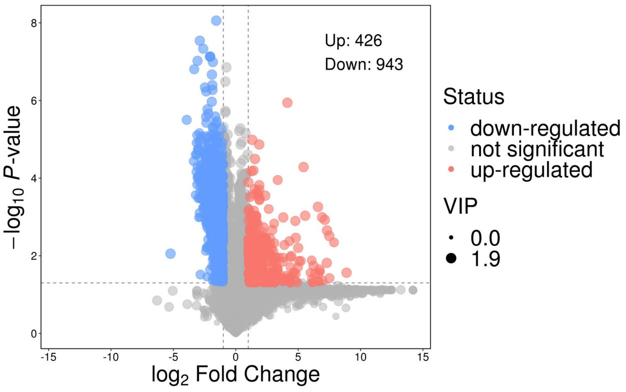


**SKS2vsSKS1**

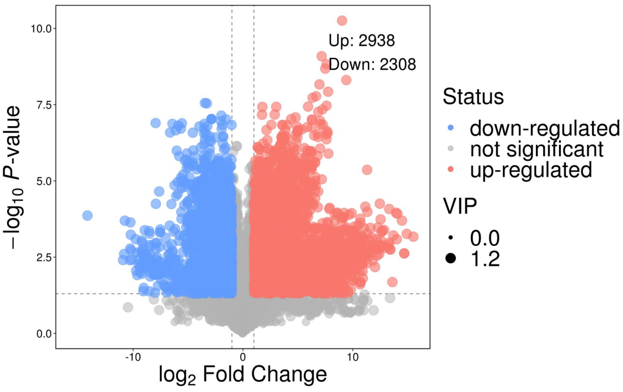


**SKS3vsSKS1**

**Fig. S10 Differential metabolite volcano plots for the four comparison groups.**

**FP represents flesh, SK represents skin, S1 represents green fruit stage, S2 represents color transformation stage, and S3 represents maturity stage.Horizontal coordinates represent the fold change for each substance in the group comparison (taking the logarithm of the base 2), vertical coordinates represent the P-value of the Student's t-test (taking the negative of the logarithm of the base 10), and the size of the scatter represents the VIP value of the OPLS-DA model, with larger scatters resulting in larger VIP values. Significantly up-regulated metabolites are shown in red, significantly down-regulated metabolites are shown in blue, and non-significantly different metabolites are gray.**

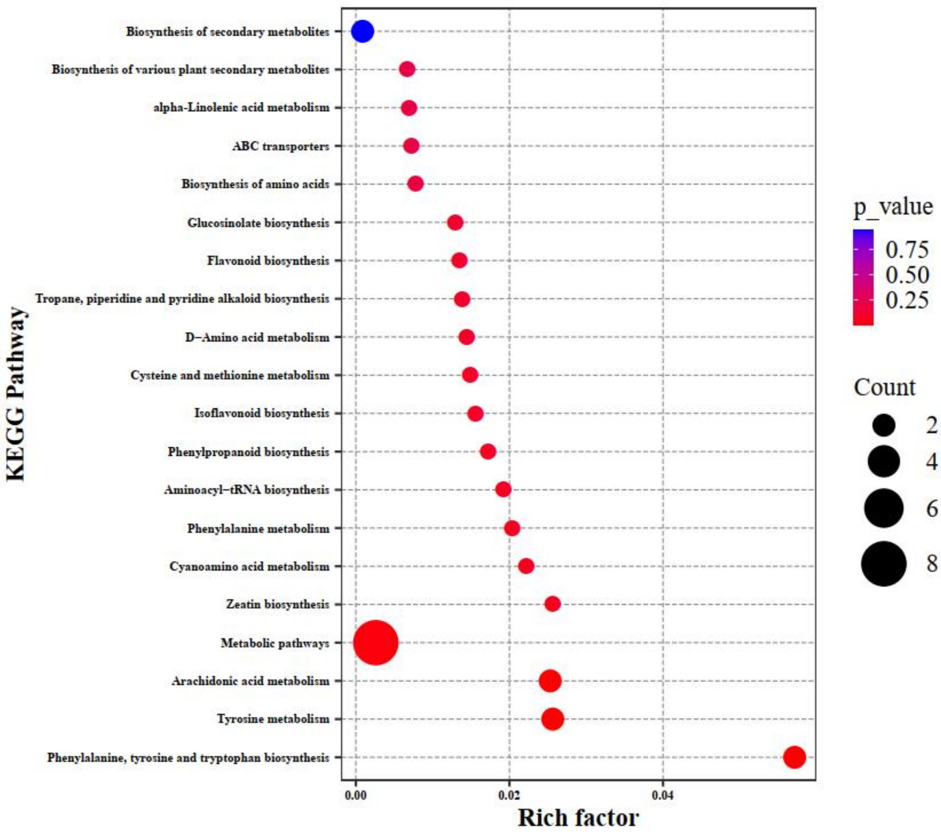


FPS2vsFPS1

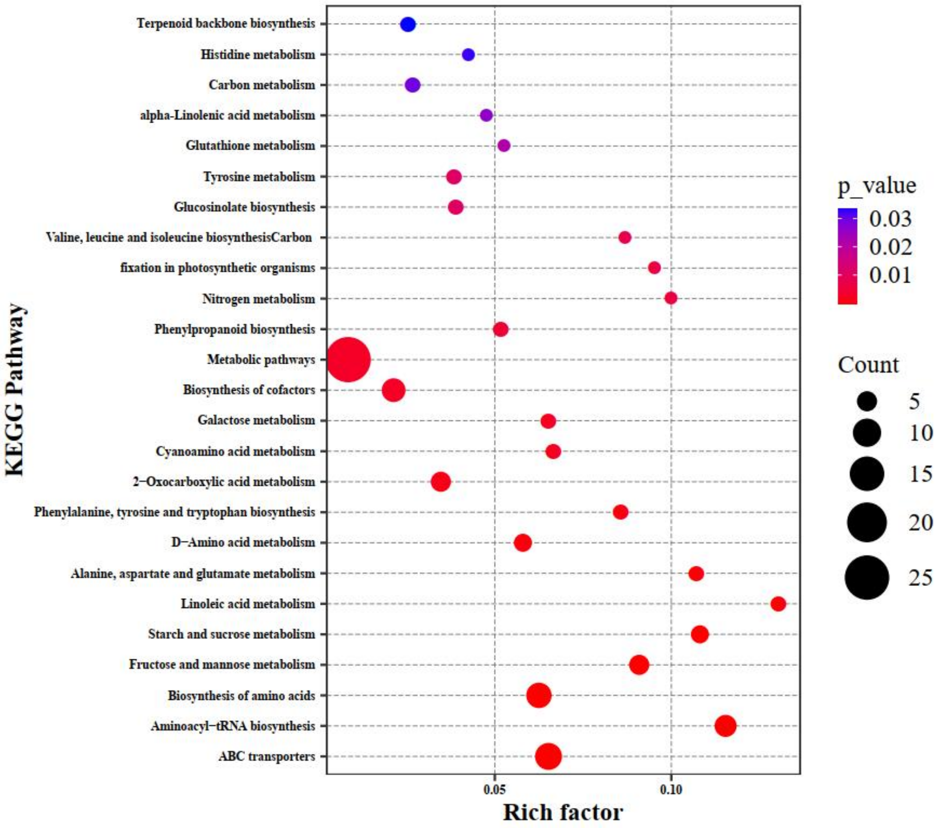


FPS3vsFPS1

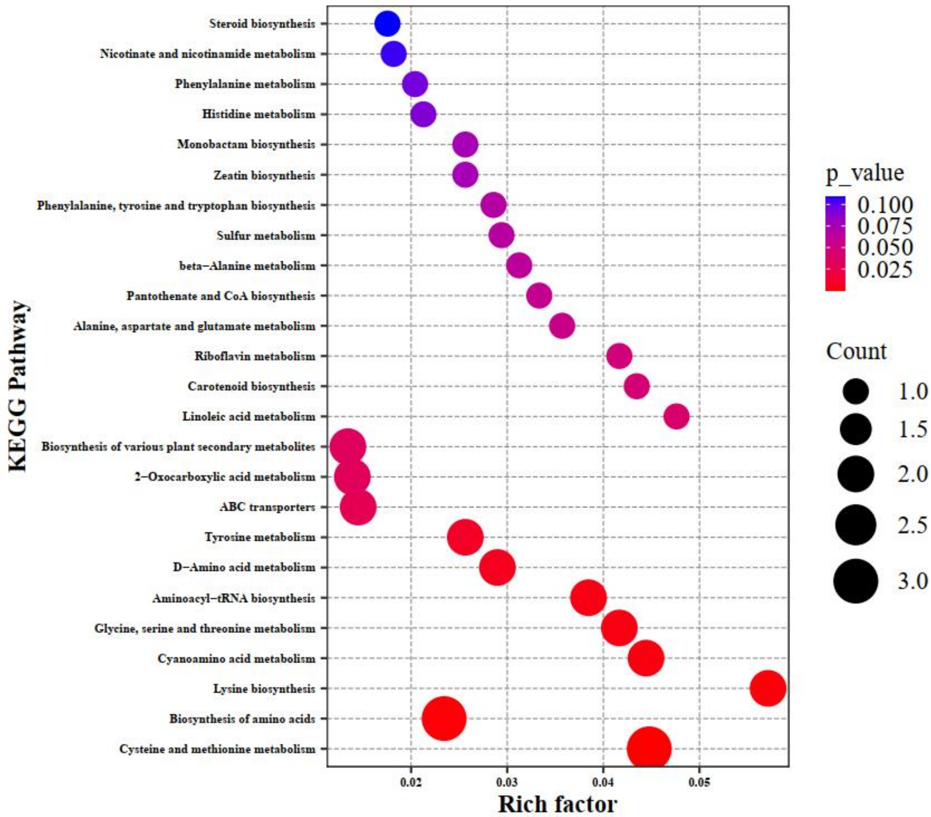


SKS2vsSKS1

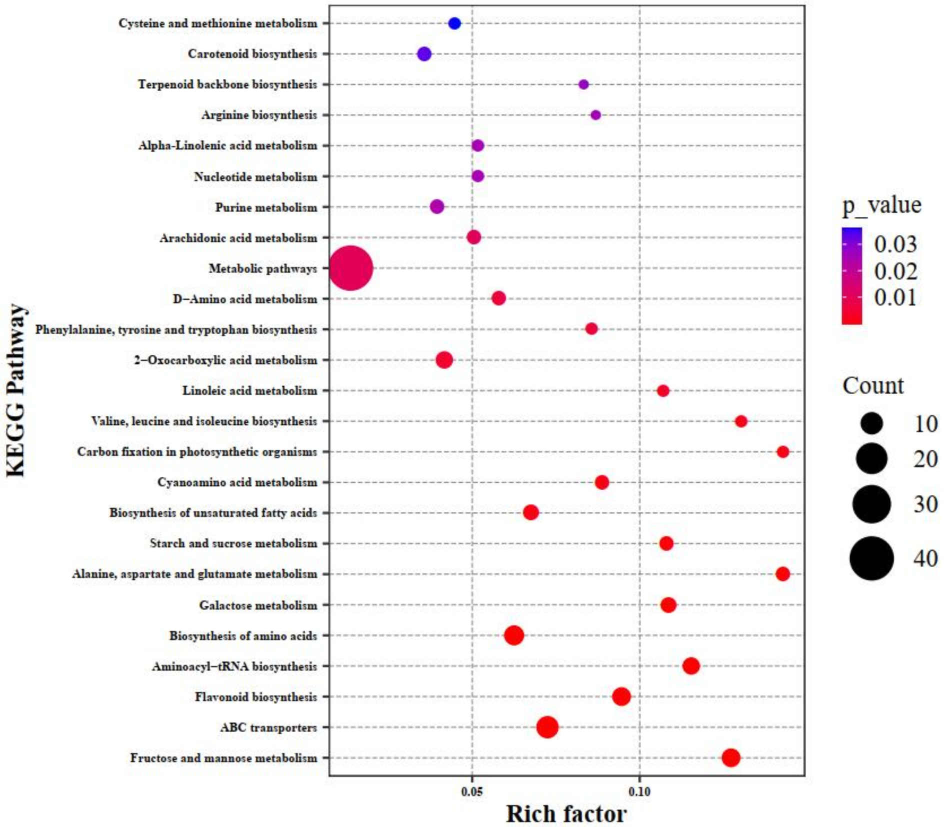


SKS3vsSKS1

**Figure S11 Differential metabolite KEGG enrichment analysis. The horizontal coordinates indicate the corresponding Rich Factor for each pathway, and the vertical coordinates are the KEGG metabolic pathway names. The size of the dots indicates the number of differential metabolites enriched in that pathway. The color indicates the size of the p-value; smaller**

**p-values and reddish colors indicate more significant enrichment.**

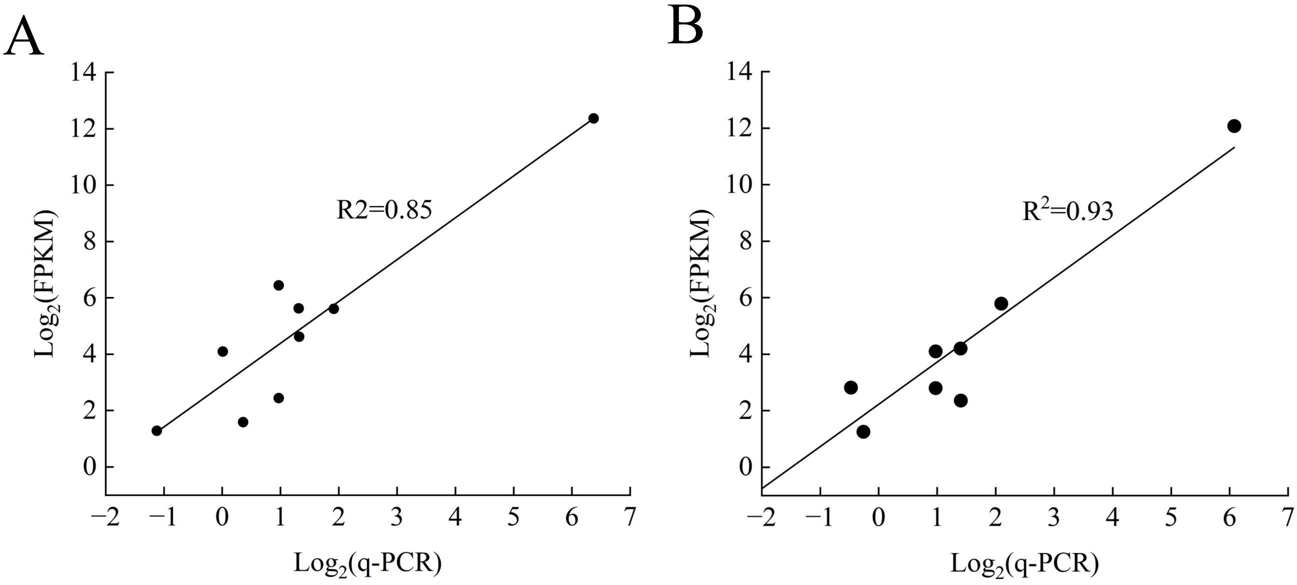


**Figure S12 Validation of qRT-PCR of genes related to the synthesis of volatile compounds at three ripening stages. A: skin ; B: pulp.**
